# Suppression of Hepatic PPARα in Primary Biliary Cholangitis Is Modulated by miR-155

**DOI:** 10.3390/cells11182880

**Published:** 2022-09-15

**Authors:** Monika Adamowicz, Agnieszka Kempinska-Podhorodecka, Joanna Abramczyk, Jesus M. Banales, Piotr Milkiewicz, Malgorzata Milkiewicz

**Affiliations:** 1 Department of Medical Biology, Pomeranian Medical University in Szczecin, 70-111 Szczecin, Poland; 2 Department of Liver and Gastrointestinal Diseases, Biodonostia Health Research Institute, Donostia University Hospital, University of the Basque Country (UPV/EHU), CIBERehd, Ikerbasque, 20014 San Sebastian, Spain; 3 Department of Biochemistry and Genetics, School of Sciences, University of Navarra, 31009 Pamplona, Spain; 4 Liver and Internal Medicine Unit, Medical University of Warsaw, 02-097 Warsaw, Poland; 5 Translational Medicine Group, Pomeranian Medical University, 70-111 Szczecin, Poland

**Keywords:** miRNA, PPARα, liver, primary biliary cholangitis

## Abstract

Background: PPARα is a ligand-activated transcription factor that shows protective effects against metabolic disorders, inflammation and apoptosis. Primary biliary cholangitis and primary sclerosing cholangitis result in the intrahepatic accumulation of bile acids that leads to liver dysfunction and damage. Small, non-coding RNAs such as miR-155 and miR-21 are associated with silencing PPARα. Methods: The expression of miR-155, miR-21 and PPARα were evaluated using real-time PCR on liver tissue, as well as on human hepatocytes (HepG2) or cholangiocytes (NHCs) following exposure to lipopolysaccharide (LPS), glycodeoxycholic acid (GCDCA), lithocholic acid (LCA) and/or ursodeoxycholic acid (UDCA). Results: A reduction of PPARα in primary biliary cholangitis (PBC) livers was associated with miR-21 and miR-155 upregulation. Experimental overexpression of either miR-155 or miR-21 inhibited PPARα in hepatocytes, whereas, in cholangiocytes, only miR-21 suppressed PPARα. Both GCDCA and LCA induced the cell type-specific upregulation of miR-155 or miR-21. In HepG2, LPS-induced miR-155 expression was blocked by a cotreatment with UDCA and was associated with PPARα upregulation. In NHC cells, the expression of miR-21 was induced by LPS but did not affect PPARα expression. Conclusions: Hepatic PPARα expression is reduced in PBC livers as a likely result of miR-155 overexpression. UDCA effectively reduced both baseline and LPS-induced miR-155 expression, thus preventing the suppression of PPARα.

## 1. Introduction

Primary biliary cholangitis (PBC) is a slow, progressive, chronic liver disease that predominantly affects middle-aged women [1]. While the aetiology of PBC has not been established, it is believed that cholangiocyte secretory failure and/or autoimmunity against intrahepatic bile ducts is linked to the presence of auto-reactive T-lymphocytes and raised plasma concentrations of specific anti-mitochondrial antibodies (AMA) [1]. Disrupted bile acid metabolism in the entero-hepatic circulation, enhanced oxidative stress, and induced inflammation cytokines causes cholestatic liver damage, which ultimately leads to liver fibrosis and cirrhosis. Another chronic cholestatic condition is primary sclerosing cholangitis (PSC), which frequently is associated with inflammatory bowel disease. Ursodeoxycholic acid (UDCA) is the first-line treatment for patients with PBC or PSC and significantly delays the progression of liver disease in the majority of cases [2].

Peroxisome proliferator-activated receptor alpha (PPARα) belongs to the superfamily of nuclear receptors (PPARs) that are ligand-activated transcription factors. PPARα regulates gene expression by binding with its heterodimeric partner, retinoid X receptor, to specific PPAR-response elements. PPARα is primarily expressed in tissues with fatty acid oxidation activity, including the liver, and regulates the expression of multiple genes involved in lipid metabolism and energy homeostasis. It is also involved in protecting against inflammation and cell apoptosis [3]. PPARα has an important role in both the inhibition of excessive inflammatory responses and in the development of innate host defences [4]. PPARα also protects against hyperglycaemia-induced endothelial inflammation and the retinal cell apoptosis pathway via blocking of the nuclear factor-kB pathway [5]. The pivotal role of PPARα in the maintenance of self-tolerance and immune homeostasis is mediated via iTregs induced by PPARα-dependent Foxp3 expression [6]. PPAR agonists (fibric acid derivatives) contribute to a range of actions, including cholesterol and bile acid (BA) homeostasis, and hinder the proinflammatory response. Both bezafibrate, an agonist of all three isoforms of human PPARs, and fenofibrate, a PPARα–selective agonist, lower serum liver biochemical markers in patients with PBC [7]. The beneficial effect of fibrates in PBC is explained by its anti-cholestatic function, as they have the ability to inhibit bile acid uptake and synthesis, as well as reduce the toxicity of bile through the translocation of phosphatidylcholine into bile [8,9]. In PBC patients who do not respond satisfactorily to UDCA treatment, the addition of bezafibrate has led to the reduction of both fibrosis and the inflammatory response [10]. Although PPARα agonists are given consideration in the treatment of various cholestatic liver disorders, knowledge of hepatic expression of PPARα at different stages of the disease is scarce.

MicroRNAs (miRs) are naturally occurring, highly conserved families of short, noncoding RNAs that regulate gene expression either via the inhibition of transcription or by repressing mRNA translation. A number of human diseases, including cancer, metabolic disorders, immune dysfunction and liver diseases, are associated with abnormal miRNA profiles [11]. A single miRNA can target numerous transcripts; therefore, the dysregulated expression of miRs can modify multiple target proteins. Some miRs, such as miR-155 or miR-21, are able to suppress the expression of different PPAR isoforms in distinct tissues [12,13]. MiR-155 was one of the earliest to be identified as a modulator of both the immune response and autoimmune development. Moreover, it appears to be the most relevant miRNA involved in several liver diseases [13,14,15]. In contrast, miR-21 is most abundantly expressed in hematopoietic cells, and its main role is in resolving inflammation and the suppression of proinflammatory responses [16,17]. Its absence gives rise to vascular inflammation and plaque formation.

In view of the critical role of PPARα signalling in the regulation of the immune response and the physiological relevance of miR-155 or miR-21 in the regulation of the PPARα gene, we evaluated the expression of these factors in liver tissue. Primary normal human cholangiocyte and hepatocyte cell lines were used to investigate: (i) whether PPARα expression is modulated by miR-155 or miR-21, (ii) the effect of toxic bile acids and LPS stimulation on miR-15 and miR-21 expression and (iii) the effect of UDCA treatment on PPARα expression.

## 2. Materials and Methods

### 2.1. Liver Tissue

Liver tissue specimens were obtained either during routine percutaneous liver biopsies from patients with early-stage (F0-F2) esPBC (*n* = 18) or were collected from explanted livers of patients with advanced (F4) PBC (*n* = 24) or primary sclerosing cholangitis (PSC) (*n* = 18) who underwent liver transplantation. Control liver samples (*n* = 16) were comprised of large-margin liver resections of colorectal metastases that showed no pathologist-identified microscopic changes indicative of liver disease. The samples were collected in the Hepatology and Internal Medicine Unit of the Medical University of Warsaw. Each patient gave informed consent prior to participating in the study. Table 1 lists the patient demographic details.

### 2.2. Cell Culture and Treatments

Primary normal human cholangiocytes (NHC), as well as the human hepatocarcinoma cell line (HepG2, American Type Culture Collection), were used for the in vitro studies [18,19]. NHC cells were established, characterised and cultured, as previously described [20,21,22]. For all analyses, NHCs and HepG2s were seeded in 6-well plates (3 × 105 cells/well) and allowed to attach overnight. Cells were transfected with commercially available miRNA Mimics for miR-155 and miR-21 (mirVana^®^ miRNA mimic hsa-miR-155; ID: MC28440; hsa-miR-21 ID: 477975_mir Ambion, Austin, TX, USA). Transient transfection was performed using Lipofectamine RNAiMAX (Invitrogen, Carlsbad, CA, USA) according to the manufacturer’s protocol. Vehicle-treated cells (Lipofectamine) were used as the control group. Forty-eight hours after transfection, the HepG2 and NHC cells were lysed and frozen as pellets for further analysis. For all experiments, UDCA (*U5127-1G*, Sigma-Aldrich, St. Louis, MO, USA) was dissolved as a 100 mM stock solution in EtOH. HepG2 and NHC cells were incubated with UDCA alone (50–200 µM) or two hours prior to 24-h stimulation with a lipopolysaccharide from *Escherichia coli* 0111:B4 (LPS, 5 µg/mL L4391-1MG SIGMA). To investigate the effect of bile acids, HepG2 and NHC cells were exposed to 500 µM of GCDCA and 150 µM GCDCA (ID: 24895023, Sigma-Aldrich, St. Louis, MO, USA), respectively. The effect of lithocholic acid (LCA) at a dose of 100 µM (LCA, Sigma-Aldrich, St. Louis, MO, USA) was tested independently in both cell types for 24 h. All experiments were repeated at least three times, and the untreated cells were used as a negative control. Cells were stored at −80 °C until molecular analyses were performed.

### 2.3. MicroRNA and mRNA Extraction and Quantification

Total RNA was extracted using the RNeasy Kit (Qiagen, Hilden, Germany) and subjected to reverse transcription using either the TaqMan Advanced miRNA cDNA Synthesis Kit (Applied Biosystems, Thermo Fisher Scientific, Waltham, MA, USA) for the quantitative analysis of microRNA or SuperScript IV RT (Invitrogen, Carlsbad, CA, USA) for further gene expression analysis according to the manufacturer’s protocol. The expression of miR-155, miR-21 and the reference miRNA miR-16-5p were measured using TaqMan^®^ Advanced miRNA Assays (Assays ID 002623_mir, 477975_mir and 477860_mir, respectively) and TaqMan^®^ Fast Advanced Master Mix (Applied Biosystems, Waltham, MA, USA). The quantitative analyses of the change in expression of specific target genes were measured using the 7500 Fast Real-Time PCR System (Applied Biosystems, Foster City, CA, USA) using human TaqMan Gene Expression Assays for PPARα (Hs00947539_m1), PDCD4 (Hs00377253_m1), PTEN (Hs02621230_s1), IL-6 (Hs001741131-m1), IL-1B (Hs01555410-m1) and 18S RNA (Hs99999901_s1). Relative amounts of transcripts in comparison to controls were determined using the 2^−ΔΔCt^ formula [23].

### 2.4. Immunoblot Analysis

Proteins were extracted from liver tissue samples by homogenisation with lysis buffer (RIPA buffer) supplemented with a protease inhibitor cocktail (Roche, Basel, Switzerland) and phosphatase inhibitors (PhosSTOP EASYpack; Roche, Basel, Switzerland). Proteins were electrophoresed on SDS-polyacrylamide gels and then blotted onto a polyvinylidene difluoride PVDF membrane (Thermo Scientific, Rockford, IL, USA) under semi-dry transfer conditions (Thermo Scientific, Rockford, IL, USA). After blocking with 5% non-fat dried milk, membranes were probed overnight at 4 °C using the primary antibodies: anti-PPARα (H-2): SC-398394, Santa Cruz Biotechnology, Inc.) followed by incubation with peroxidase-conjugated secondary anti-mouse (1:1000) antibodies (GE Healthcare, code: NA9310). Protein loading was normalised to anti-GAPDH (1:5000, sc-25,778 + HRP; Santa Cruz). Bands were visualised through a chemiluminescence detection system (Chemiluminescent HRP Substrate, Millipore, MA, USA) and quantified using the MicroChemi 2.0 System and GelQuant software (Maale HaHamisha, Jerusalem, Israel).

### 2.5. Immunohistochemistry

Immunohistochemical analyses of liver sections were performed using the ImmPRESS Universal Reagent kit (Vector Laboratories, Burlingame, CA, USA, #SP-2001). The deparaffinisation of the tissue sections were followed by antigen unmasking with antigen retrieval buffer (citrate-based solution, pH 6.0; 95 °C for 20 min). After blocking with ready-to-use normal horse serum (2.5%), samples were incubated with primary antibodies against PPARα (sc-398394, Santa Cruz Biotechnology, Inc. Oregon, USA) for 90 min in room temperature. After washing, samples were left for 30 min with ImmPRESS reagent and then dyed with a substrate/chromogen mixture (ImmPACT™ DAB). After washing, samples were counterstained with haematoxylin and mounted (Aqueous Permanent Medium, Dako, Denmark). A Zeiss Axio Imager Z2 optical microscope equipped with the Zen Pro 2011 acquisition program was used to acquire the images.

### 2.6. Statistical Analysis

StatView software version 5.0 (SAS Institute, Cary, NC, USA) was used for the statistical analyses. Statistical differences between groups were analysed using the Student’s *t*-test and multiple groups’ comparisons were performed with one-way analysis of variance (ANOVA). All graphs were generated using GraphPad Prism version 7 software (GraphPad Software, San Diego, CA, USA). Data are expressed as the mean ± SEM. Results were considered statistically significant when *p*-values were less than 0.05.

## 3. Results

**Figure 1:**
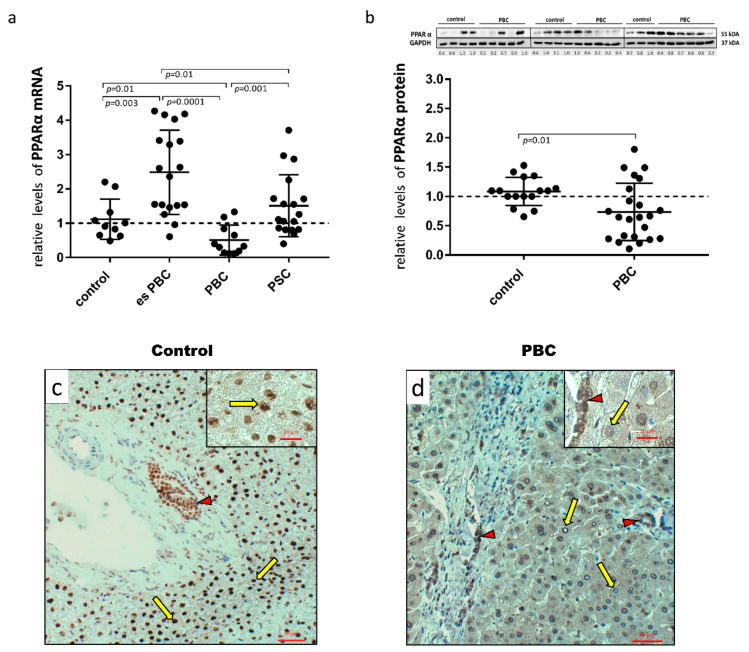
Presence of PPARα in liver tissue samples obtained from the controls, the early-stages of primary biliary cholangitis (esPBC), cirrhotic PBC and primary sclerosing cholangitis (PSC) patients. PPARα mRNA expression was suppressed in cirrhotic PBC (**a**), and Western blot analysis confirmed lower levels of PPARα at the protein level (**b**). Levels of gene expression were normalised to the endogenous reference, 18S RNA and the levels of each protein were normalised to GAPDH. Dots illustrate each patient, and the data are presented as mean plus interquartile range (IQR). Statistical analysis was performed using ANOVA or a Student’s *t*-test. Immunohistochemical staining clearly showed a dominant nuclear localisation of the PPARα protein in the control tissue (**c**) in contrast to liver tissue from patients with PBC (**d**). Both hepatocytes (yellow arrows) and cholangiocytes (red arrow heads) were positive for PPARα. Original magnification 200× or 400× (inserts).

First, human hepatic samples obtained from patients with PBC or PSC during liver transplantation were examined for PPARα expression. The analysis showed a marked reduction both at the mRNA (Figure 1a) and protein (Figure 1b) levels in PBC livers. The 50% reduction in mRNA expression was significant in comparison to the controls (*p* = 0.01), early-stage esPBC (*p* = 0.01) and in comparison to another cholestatic liver disease, PSC (*p* = 0.001; Figure 1a). A histological evaluation of PPARα in the control (Figure 1c) and PBS livers (Figure 1d) demonstrated the expression of this protein both in hepatocytes and cholangiocytes within bile ducts. However, in contrast to PBC livers, in the control tissue, a strong nuclear localisation of PPARα within hepatocytes was observed.

**Figure 2:**
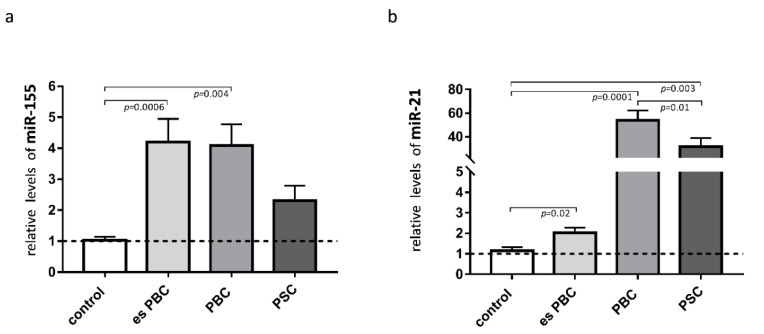
Expression of miR-155 and miR-21 in liver tissue. In patients with primary biliary cholangitis (PBC), both miR-155 (**a**) and miR-21 (**b**) expression were increased in comparison to the healthy controls. In livers of PSC patients, only miR-21 was substantially induced. MiR-16 served as the reference for loading. Bars indicate the mean ± SEM. Statistical analysis was performed using ANOVA or a Student’s *t*-test.

Knowing that both miR-155 and miR-21 target PPARα, we estimated the levels of these miRs in the liver tissue. The observed phenomenon of the reduction of PPARα in cirrhotic PBC livers was associated with a substantial induction of miR-155 (3.5-fold increase vs. controls, *p* = 0.004; Figure 2a) and miR-21 (50-fold increase vs. controls, *p* = 0.0001, and *p* = 0.01 vs. PSC; Figure 2b). There was a negative correlation between PPARα and miR-21 (r = −0.45, *p* = 0.01), and the expression of miR-21 positively correlated with the inflammatory cytokines, both IL-6 (r = 0.56, *p* = 0.003) and IL-1b (r = 0.46, *p* = 0.027).

**Figure 3:**
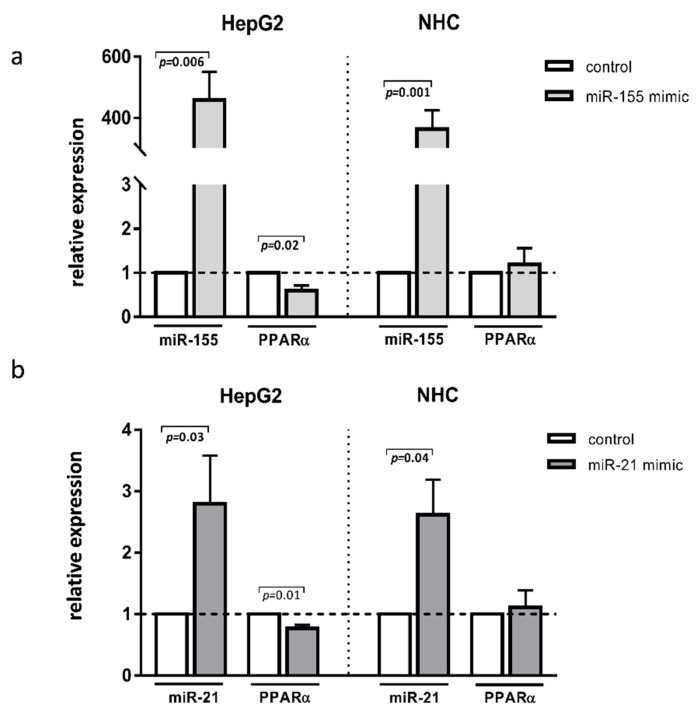
PPARα, expression after miR-155 or miR-21 activation. Human hepatocarcinoma (HepG2) and normal human cholangiocyte (NHC) cells were transfected with miR-155 mimic (**a**) or miR-21 mimic (**b**). Increased levels of these miRNAs were confirmed in both cell lines. Overexpression of both miR-155 and miR-21 led to the strong downregulation of PPARα in HepG2 but not in NHC cells. Each experiment was repeated at least three times. Levels of gene expression were normalised to the reference miR-16 for miRNA or 18S RNA for other genes. Bars indicate the mean ± SEM. Student’s *t*-test was used for the quantitative data analysis.

To further investigate the specific role of these miRNAs in the aforementioned liver diseases, we transfected HepG2 and NHC cells with either miR-155 or miR-21 mimics. The experimental overexpression of miR-155 reduced the PPARα mRNA levels in HepG2 cells (0.6 ± 0.01 vs. 1.0 ± in the controls *p* = 0.02; Figure 3a) but not in NHC cells (Figure 3a). Similarly, the overexpression of miR-21 inhibited PPARα in HepG2 (0.8 ± 0.05. vs. 1.0 ± in the controls *p* = 0.01 Figure 3b) but not in NHC cells (Figure 3b).

**Figure 4:**
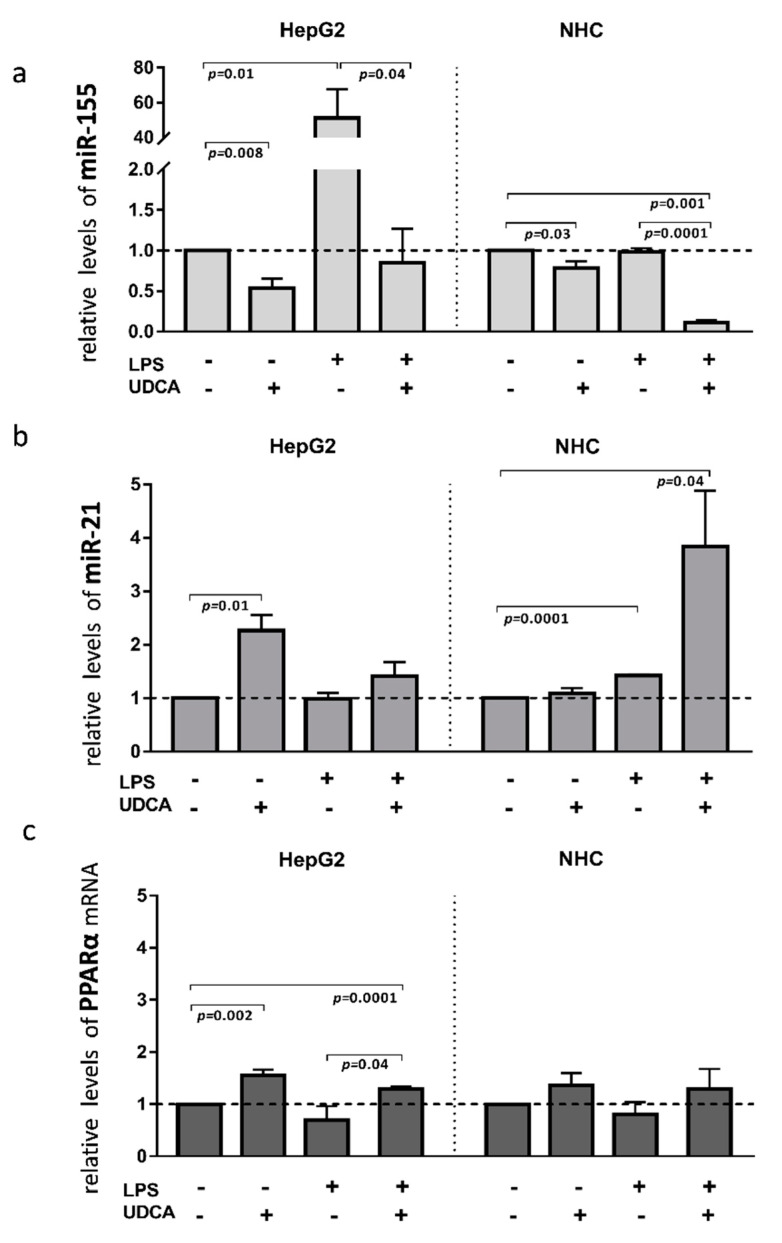
The effect of lipopolysaccharide (LPS) and/or ursodeoxycholic acid (UDCA) exposure in human hepatocarcinoma (HepG2) and normal human cholangiocyte (NHC) cell lines. LPS stimuli enhanced miR-155 (**a**) in HepG2 cells and miR-21 (**b**) in NHC cells. MiR-155 was enhanced after the incubation of HepG2 cells with LPS, whereas, in NHC, the expression of miR-21 was induced by LPS stimuli. UDCA reduced both the baseline and LPS-induced miR-155 expression in HepG2 cells, which was accompanied by the upregulation of PPARα. In NHC cells, UDCA enhanced the expression of miR-21 but did not affect PPARα expression (**c**). Bars indicate the mean ± SEM. The statistical analysis was performed using ANOVA.

Inflammation contributes to the pathogenesis of PBC; therefore, to investigate the effect of activated inflammatory response on the miRs expression, we exposed HEPG2 and NHC cells to lipopolysaccharide (LPS), which activates Toll-like receptor 4 (TLR4). The incubation of HepG2 cells with LPS led to the induction of miR-155 expression (40-fold, *p* = 0.001 vs. controls, Figure 4a), which was blocked by the UDCA cotreatment (*p* = 0.04 vs. LPS). Moreover, UDCA alone suppressed the baseline expression of miR-155 (*p* = 0.008 vs. controls) in HepG2. In contrast, in NHC cells, miR-155 expression did not change after LPS exposure, but, similarly to HepG2, UDCA substantially reduced both the baseline expression of miR-155 (20% reduction, *p* = 0.03 vs. nontreated control cells) and after LPS exposure (80% reduction, *p* = 0.001 vs. nontreated cells; Figure 4a). The expression of miR-21 was induced by LPS only in NHC cells (1.4-fold increase, *p* = 0.0001 vs. nontreated cells) and was further enhanced by the UDCA cotreatment (4-fold increase, *p* = 0.04 vs. nontreated cells; Figure 4b). *PPARα* gene expression was enhanced by UDCA in both LPS-stimulated (*p* = 0.0001 vs. nontreated cells; Figure 4c), and non-LPS-stimulated HepG2 cells (*p* = 0.002 vs. nontreated cells; Figure 4c).

**Figure 5:**
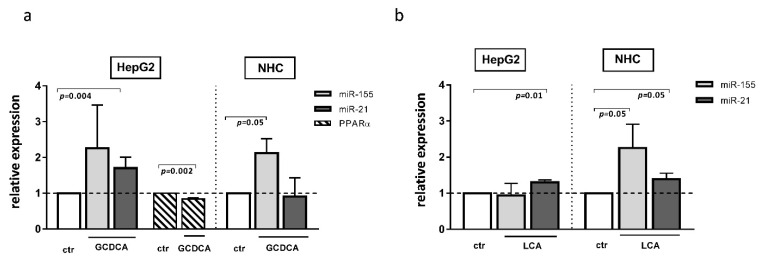
The effect of glycochenodeoxycholic acid (GCDCA) and lithocholic acid (LCA) in human hepatocarcinoma (HepG2) and normal human cholangiocytes (NHC) cells. In HepG2 cells, both GCDCA (**a**) and LCA (**b**) induced miR-21. In NHC cells, both bile acids induced miR-155, whereas miR-21 was enhanced only after LCA stimulation. The PPARα level was reduced in HepG2 after GCDCA treatment (**a**). Each experiment was repeated at least three times. MiR-16 was used as an endogenous reference for miRNA or 18S RNA for other genes. Bars indicate the mean ± SEM. A Student’s *t*-test were used for a quantitative data analysis.

The development of cholestatic liver diseases such as PBC is negatively impacted not only by inflammation but also by chronic exposure to toxic bile acid. There have been few studies on the effect of bile acids on miR profiles. It was reported that, in primary human hepatocytes, chenodeoxycholic acid affected the expression of different miRs; however, neither miR-155 or miR-21 were evaluated in the study [24]. Our study showed that both glycochenodeoxycholic acid (GCDCA) and lithocholic acid (LCA) induced miR-21 expression in HepG2 cells (1.7 ± 0.15, *p* = 0.004 and 1.3 ± 0.06, *p* = 0.01, respectively, Figure 5a,b), whereas, in NHC cells, these bile acids upregulated miR-155 (2.13 ± 0.39, *p* = 0.05 and 2.253 ± 0.66, *p* = 0.05, respectively, Figure 5a,b). Moreover, in NHC cells, the expression of miR-21 was stimulated by LCA exposure (Figure 5b). Interestingly, one of these bile acids species, namely GCDCA, decreased the PPARα level but only in the HepG2 cell line (0.84 ± 0.03, *p* = 0.002, Figure 5a).

## 4. Discussion

This study provides new insight into the regulation of PPARα in cholestatic livers. In cirrhotic PBC livers, a substantial reduction of PPARα expression was associated with the upregulation of both miR-21 and miR-155. A cell-based analysis demonstrated that the experimental overexpression of either miR-155 or miR-21 inhibited *PPARα* mRNA in hepatocytes, whereas, in cholangiocytes, only the overexpression of miR-21 led to *PPARα* downregulation. The factors responsible for the induction of these miRNAs appeared to be cell type-specific when the HepG2 and NHC cell lines were compared. Moreover, a new biological function of UDCA as a modulator of miR-21 and miR-155 in those cells was found.

We observed a substantial reduction of both the mRNA and protein levels of PPARα in cirrhotic PBC livers. In contrast, in another cholestatic condition such as PSC, the hepatic expression of this nuclear receptor was comparable to the control values. Moreover, this study showed that the inhibition of PPAR was present only in advanced phases and not in the early stages of the disease. This is in line with reports on altered hepatic PPARα expression in liver diseases. In patients with Wilson’s disease, PPARα expression was found to be altered in proportion to the progression of liver injury, i.e., it was enhanced in patients with mild liver impairment but reduced in patients with moderate or intense liver damage [25]. Similarly, in subjects with non-alcoholic steatohepatitis (NASH), hepatic PPARα expression declined with the development of NASH features and was negatively correlated with the severity of steatosis, hepatocyte ballooning or fibrosis [26]. In the context of cholestatic liver diseases, our study represents a novel report, as there is a lack of information on the hepatic expression of this nuclear expression under conditions of sustained cholestasis.

PPARα plays a crucial role in bile acid homeostasis via the regulation of bile acid biosynthesis, transport and secretion [27]. Furthermore, it has been demonstrated that fenofibrate-activated PPARα signalling eliminates oxidative stress and attenuates cholestatic liver injury [28]. In patients with PBC, therapies based on PPARα agonists are well-tolerated and allied with a significant decrease in the alkaline phosphatase (ALP) levels and anti-inflammatory markers [29,30]. Moreover, a prospective, long-term, longitudinal study showed a potentially beneficial effect of bezafibrate in combination with UDCA in patients who had an inadequate response to UDCA [10]. A key factor influencing the effectiveness of fibrate-based therapy is the adequate expression of PPARα; however, our study showed a substantial reduction of PPARα expression in cirrhotic PBC livers. Therefore, understanding the molecular mechanism responsible for the hepatic reduction of PPARα is of particular importance.

This study focused on two miRNAs that are known to modulate the *PPARα* gene, i.e., miR-155 and miR-21. We showed that, in cirrhotic PBC livers, the expressions of both miRNAs were substantially upregulated, which is in contrast to cirrhotic PSC, where only miR-21 was increased. MiR-155 modifies proinflammatory responses that affect not only immune cells but also hepatic parenchymal cells, including hepatocytes. MiR-155 is known to exert pleiotropic functions depending on the aetiology and disease context. However, miR-155 expression in cholestatic diseases has not been described to date, although there are reports from other hepatobiliary diseases. The serum level of miR-155 is increased in patients with alcoholic cirrhosis [13] and in non-alcoholic fatty liver disease (NAFLD) [31]. In patients with biliary atresia or PBC, an inverse correlation between miR-155 and the suppressor of cytokine signalling 1 (SOCS1) has been reported [32,33]. In addition, the antiviral treatment of hepatitis C (HCV) patients normalised the level of miR-155 in peripheral monocytes in contrast to non-responders [14].

Since the specific suppression of PPARα in PBC livers was accompanied by a substantial upregulation of miR-155 and miR-21, we conducted functional studies in hepatocyte and cholangiocyte cells. Of note, the experimental overexpression of either miR-155 or miR-21 suppressed the PPARα levels in HepG2 cells but not in NHC cells. The downregulation of PPARα by miR-21 has been described in numerous pathologic processes. For example, miR-21 directly inhibits PPARα translation [12,34], which promotes the expression of vascular cell adhesion molecule-1 (VCAM-1) and favours the adhesion of inflammatory cells [35] or leads to retinal microvascular dysfunction [36]. In the context of liver pathology, PPARα was demonstrated to be a direct target of miR-155 or miR-21 in mouse biliary, hepatic and inflammatory cells in a mouse model of alcohol-induced steatohepatitis, NASH and in the development of hepatocellular carcinoma (HCC) [13,34,35,37].

Further, to investigate the factors involved in the hepatic upregulation of these miRNAs, we induced inflammatory responses by LPS or exposed the cells to toxic bile acids. The factors involved in the hepatic upregulation of the miRNAs were cell-specific. Consistent with previous studies [38,39], we found that miR-155 expression was induced by the inflammatory response activated by LPS exposure but only in HepG2 cells. In NHC cells, the induction of this miRNA was only observed in response to toxic bile acids (both GCDA and LCA). Comparatively little is known about the effects of bile acids on the cellular microRNAome. In accordance with our observations, chenodeoxycholic acid did not affect miR-155 expression in primary human hepatocytes [24]; however, the acidic, bile-induced upregulation of miR-155 was noted in human hypopharyngeal primary cells [40]. Here, we present the first report that prolonged exposure to bile acids may induce miR-155 expression in normal human cholangiocytes.

Interestingly, LPS-induced miR-155 expression in HepG2 was overridden by UDCA treatment. Moreover, UDCA effectively reduced the baseline expression of miR-155 in both HepG2 and NHC cells. Given the relevance of miR-155 dysregulation in the proper homeostasis of the immune response and macrophage polarisation [41], this is a noteworthy observation that confirms the positive role of UDCA in modulating miR-155 expression. Previously, UDCA was shown to effectively decrease both miR-34 in primary rat hepatocytes and miR-122 in human serum [42,43].

Considerable evidence has highlighted miR-21 as one of the key switches that controls the magnitude of inflammation [16,44]. However, its presence is not entirely attributed to a proinflammatory or an immunosuppressive condition. Recently, miR-21 has been proposed as a negative modulator of Toll-Like Receptor 4 (TLR4) signalling by targeting PTEN and PDCD4, which resulted in the elevated production of IL-10 [44,45]. Moreover, miR-21 influences the fine balance between Th1 and Th2 responses, and elevated miR-21 expression leads to a reduction of IL-6 secretion and the induction of IL-10 production in macrophages [46,47,48]. Similarly, a negative regulation of the TNF-a levels by miR-21 has been reported [16,44,48]. Thus, miR-21 dysregulation that has been observed in a number of inflammatory diseases promotes an anti-inflammatory, immunosuppressive environment. The absence of miR-21 in hematopoietic cells also enhances vascular inflammation and atherosclerosis [17]. Interestingly, we found a substantially induced expression of miR-21 in the liver tissue of both PBC and PSC patients. miR-21 was previously implicated in the development of fibrosis; however, in this study, a small increase was observed in the early stages of PBC (F0-F2), followed by a substantial increase in cirrhotic PBC. There was no correlation between the levels of miR-21 and the stages of fibrosis, which is in agreement with previous reports [49,50]. Even though miR-21 ablation has been shown to protect from fibrosis and acute oxidative stress in the livers of mice with bile duct ligation, it was an acute model of cholestasis, which did not completely mimic the sustained cholestasis that occurs in PBC [51]. Interestingly, the authors noticed that miR-21−/− mice displayed an increased hepatic TLR4 expression, which was attributed to the anti-inflammatory function of miR-21 [51].

Our current study demonstrated that LPS, and two distinct bile acid species (LCA and GCDA) induced miR-21 expression; however, the responses were cell-dependent. Consequently, the upregulation of miR-21 was observed in NHC cells after LPS or LCA incubation. This was in contrast to HepG2 cells, where GCDA and LCA exposure triggered miR-21 induction. Previously, LPS was reported to induce miR-21 in a number of cell lines, including human biliary epithelial cells and hepatic stellate cells [52,53]. In contrast to our study, an inhibition of miR-21 by another bile acid, cytotoxic deoxycholic acid (DCA) in a dose-dependent manner was reported in primary rat hepatocytes [54]. Interestingly, we noticed a further upregulation of miR-21 by UDCA in NHC cells incubated with LPS, which is in line with the observation that UDCA is a strong inducer of miR-21 in regenerating rat livers and cultured HepG2 cells [54,55]. The induction of miR-21 in murine macrophages via a treatment with LPS was associated with silencing its target genes, PTEN and PDCD4, which are powerful inhibitors of the AP-1 transcription factor [44]. In this study, the forced overexpression of miR-21 in HepG2 cells decreased PDCD4 and PTEN mRNA expressions (Figure S1). Bile acids are strong modulators of AP-1 activity, and the increase of miR-21 expression stimulated by UDCA was shown to inhibit the activation of AP-1 and thus favour a pro-proliferative environment [56].

Cumulatively, hepatic PPARα expression is substantially reduced in PBC livers, potentially as a result of enhanced miR-155 expression. Furthermore, the increased miR-21 expression in PBC and PSC livers may be implicated in resolving inflammation. UDCA effectively reduced both baseline and lipopolysaccharide-induced miR-155 expression, which prevented the suppression of PPAR.

## Figures and Tables

**Table 1:** Demographic and laboratory features of all analysed subjects.

	Control (*n* = 16)	esPBC (*n* = 18)	PBC (*n* = 24)	PSC (*n* = 18)
Gender (Female/Male)	7/9	18/0	22/2	6/12
Age (years)	50 (25–60)	55 (28–64)	57 (36–69)	33 (20–57)
Bilirubin (mgL/dL, NR: 0.1–1.1)	0.5 (0.2–1.0)	0.6 (0.3–7.8)	4.4 (0.6–21)	2.7 (0.4–32.2)
ALP (IU/L, NR: 40–120)	24 (40–118)	178 (47–456)	400 (119–1373)	387 (114–2181)
AST (IU/L, NR: 5–35)	23 (9–34)	40 (13–182)	104 (51–295)	99 (24–500)

Median and range values (in parentheses). Abbreviations: esPBC, early-stage primary biliary cholangitis; PBC, primary biliary cholangitis; PSC, primary sclerosing cholangitis; ALP, alkaline phosphatase; AST, aspartate aminotransferase and NR, normal range.

## Data Availability

Not applicable.

## Supplementary Materials

The following supporting information can be downloaded at: https://www.mdpi.com/article/10.3390/cells11182880/s1, Figure S1: Programmed cell death 4 (PDCD4) and phosphatase and tensin homologue (PTEN) expression after miR-21 activation.

## References

[1] Lleo A., Wang G.Q., Gershwin M.E., Hirschfield G. (2020). Primary biliary cholangitis. Lancet.

[2] Corpechot C., Abenavoli L., Rabahi N., Chretien Y., Andreani T., Johanet C., Chazouilleres O., Poupon R. (2008). Biochemical response to ursodeoxycholic acid and long-term prognosis in primary biliary cirrhosis. Hepatology.

[3] Ghonem N.S., Assis D.N., Boyer J.L. (2015). Fibrates and cholestasis. Hepatology.

[4] Christofides A., Konstantinidou E., Jani C., Boussiotis V.A. (2021). The role of peroxisome proliferator-activated receptors (PPAR) in immune responses. Metabolism.

[5] Hu Y., Chen Y., Ding L., He X., Takahashi Y., Gao Y., Shen W., Cheng R., Chen Q., Qi X. (2013). Pathogenic role of diabetes-induced PPAR-+- down-regulation in microvascular dysfunction. Proc. Natl. Acad. Sci. USA.

[6] Lei J., Hasegawa H., Matsumoto T., Yasukawa M. (2010). Peroxisome proliferator-activated receptor alpha and gamma agonists together with TGF-beta convert human CD4+. J. Immunol..

[7] Corpechot C., Chazouilleres O., Rousseau A. (2018). Bezafibrate in primary biliary cholangitis. N. Engl. J. Med..

[8] Grigorian A.Y., Mardini H.E., Corpechot C., Poupon R., Levy C. (2015). Fenofibrate is effective adjunctive therapy in the treatment of primary biliary cirrhosis: A meta-analysis. Clin. Res. Hepatol. Gastroenterol..

[9] Gallucci G.M., Trottier J., Hemme C., Assis D.N., Boyer J.L., Barbier O., Ghonem N.S. (2021). Adjunct fenofibrate up-regulates bile acid glucuronidation and improves treatment response for patients with cholestasis. Hepatol. Commun..

[10] Sorda J.A., Gonzalez B.E., Barreyro F.J., Avagnina A., Carballo P., Paes de Lima A., Daruich J. (2021). Bezafibrate therapy in primary biliary cholangitis refractory to ursodeoxycholic acid: A longitudinal study of paired liver biopsies at 5 years of follow up. Aliment. Pharmacol. Ther..

[11] Szabo G., Bala S. (2013). MicroRNAs in liver disease. Nat. Rev. Gastroenterol. Hepatol..

[12] Kida K., Nakajima M., Mohri T., Oda Y., Takagi S., Fukami T., Yokoi T. (2011). PPARalpha is regulated by miR-21 and miR-27b in human liver. Pharm. Res..

[13] Bala S., Csak T., Saha B., Zatsiorsky J., Kodys K., Catalano D., Satishchandran A., Szabo G. (2016). The pro-inflammatory effects of miR-155 promote liver fibrosis and alcohol-induced steatohepatitis. J. Hepatol..

[14] Bala S., Tilahun Y., Taha O., Alao H., Kodys K., Catalano D., Szabo G. (2012). Increased microRNA-155 expression in the serum and peripheral monocytes in chronic HCV infection. J. Transl. Med..

[15] Hartmann P., Tacke F. (2016). Tiny RNA with great effects: miR-155 in alcoholic liver disease. J. Hepatol..

[16] Sheedy F.J. (2015). Turning 21: Induction of miR-21 as a key switch in the inflammatory response. Front. Immunol..

[17] Canfran-Duque A., Rotllan N., Zhang X., Fernandez-Fuertes M., Ramirez-Hidalgo C., Araldi E., Daimiel L., Busto R., Fernandez-Hernando C., Suarez Y. (2017). Macrophage deficiency of miR-21 promotes apoptosis, plaque necrosis, and vascular inflammation during atherogenesis. EMBO Mol. Med..

[18] Weerachayaphorn J., Amaya M.J., Spirli C., Chansela P., Mitchell-Richards K.A., Ananthanarayanan M., Nathanson M.H. (2015). Nuclear factor, erythroid 2-like 2 regulates expression of type 3 inositol 1,4,5-trisphosphate receptor and calcium signaling in cholangiocytes. Gastroenterology.

[19] Kilanczyk E., Ruminkiewicz D., Banales J.M., Milkiewicz P., Milkiewicz M. (2022). DHEA protects human cholangiocytes and hepatocytes against apoptosis and oxidative stress. Cells.

[20] Urribarri A.D., Munoz-Garrido P., Perugorria M.J., Erice O., Merino-Azpitarte M., Arbelaiz A., Lozano E., Hijona E., Jimenez-Aguero R., Fernandez-Barrena M.G. (2014). Inhibition of metalloprotease hyperactivity in cystic cholangiocytes halts the development of polycystic liver diseases. Gut.

[21] Banales J.M., Sáez E., Uriz M., Sarvide S., Urribarri A.D., Splinter P., Bogert P.S.T., Bujanda L., Prieto J., Medina J.F. (2012). Upregulation of mir-506 leads to decreased AE2 expression in biliary epithelium of patients with primary biliary cirrhosis. Hepatology.

[22] De Urturi D.S., Buque X., Porteiro B., Folgueira C., Mora A., Delgado T.C., Prieto-Fernandez E., Olaizola P., Gomez-Santos B., Apodaka-Biguri M. (2022). Methionine adenosyltransferase 1a antisense oligonucleotides activate the liver-brown adipose tissue axis preventing obesity and associated hepatosteatosis. Nat. Commun..

[23] Applied Biosystems (2008). Guide to performing relative quantitation of gene expression using real-time quantitative PCR. Rev. Biol..

[24] Krattinger R., Bostrom A., Lee S.M.L., Thasler W.E., Schioth H.B., Kullak-Ublick G.A., Mwinyi J. (2016). Chenodeoxycholic acid significantly impacts the expression of miRNAs and genes involved in lipid, bile acid and drug metabolism in human hepatocytes. Life Sci..

[25] Nagasaka H., Miida T., Inui A., Inoue I., Tsukahara H., Komatsu H., Hiejima E., Fujisawa T., Yorifuji T., Hiranao K. (2012). Fatty liver and anti-oxidant enzyme activities along with peroxisome proliferator-activated receptors gamma and alpha expressions in the liver of Wilson’s disease. Mol Genet. Metab..

[26] Francque S., Verrijken A., Caron S., Prawitt J., Paumelle R., Derudas B., Lefebvre P., Taskinen M.R., Van H.W., Mertens I. (2015). PPARalpha gene expression correlates with severity and histological treatment response in patients with non-alcoholic steatohepatitis. J. Hepatol..

[27] Li F., Patterson A.D., Krausz K.W., Tanaka N., Gonzalez F.J. (2012). Metabolomics reveals an essential role for peroxisome proliferator-activated receptor alpha in bile acid homeostasis. J. Lipid Res..

[28] Zhao Q., Yang R., Wang J., Hu D.D., Li F. (2017). PPARalpha activation protects against cholestatic liver injury. Sci. Rep..

[29] Carrion A.F., Lindor K.D., Levy C. (2021). Safety of fibrates in cholestatic liver diseases. Liver Int..

[30] Schattenberg J.M., Pares A., Kowdley K.V., Heneghan M.A., Caldwell S., Pratt D., Bonder A., Hirschfield G.M., Levy C., Vierling J. (2021). A randomized placebo-controlled trial of elafibranor in patients with primary biliary cholangitis and incomplete response to UDCA. J. Hepatol..

[31] Longchamps R.J., Abey S.K., Martino A.C., Henderson W.A. (2014). Letter: Gender-associated cell-free microRNA profiles in non-alcoholic fatty liver disease. Aliment. Pharmacol. Ther..

[32] Kempinska-Podhorodecka A., Milkiewicz M., Wasik U., Ligocka J., Zawadzki M., Krawczyk M., Milkiewicz P. (2017). Decreased expression of vitamin D receptor affects an immune response in primary biliary cholangitis via the VDR-miRNA155-SOCS1 pathway. Int. J. Mol. Sci..

[33] Zhao R., Dong R., Yang Y., Wang Y., Ma J., Wang J., Li H., Zheng S. (2017). MicroRNA-155 modulates bile duct inflammation by targeting the suppressor of cytokine signaling 1 in biliary atresia. Pediatr. Res..

[34] Loyer X., Paradis V., Henique C., Vion A.C., Colnot N., Guerin C.L., Devue C., On S., Scetbun J., Romain M. (2016). Liver microRNA-21 is overexpressed in non-alcoholic steatohepatitis and contributes to the disease in experimental models by inhibiting PPARalpha expression. Gut.

[35] Zhou J., Wang K.C., Wu W., Subramaniam S., Shyy J.Y., Chiu J.J., Li J.Y., Chien S. (2011). MicroRNA-21 targets peroxisome proliferators-activated receptor-alpha in an autoregulatory loop to modulate flow-induced endothelial inflammation. Proc. Natl. Acad. Sci. USA.

[36] Chen Q., Qiu F., Zhou K., Matlock H.G., Takahashi Y., Rajala R.V.S., Yang Y., Moran E., Ma J.X. (2017). Pathogenic role of microRNA-21 in diabetic retinopathy through downregulation of PPARalpha. Diabetes.

[37] Koenig A.B., Barajas J.M., Guerrero M.J., Ghoshal K. (2018). A comprehensive analysis of argonaute-CLIP data identifies novel, conserved and species-specific targets of miR-21 in human liver and hepatocellular carcinoma. Int. J. Mol. Sci..

[38] Bala S., Marcos M., Kodys K., Csak T., Catalano D., Mandrekar P., Szabo G. (2011). Up-regulation of microRNA-155 in macrophages contributes to increased tumor necrosis factor {alpha} (TNF{alpha}) production via increased mRNA half-life in alcoholic liver disease. J. Biol. Chem..

[39] Csak T., Bala S., Lippai D., Kodys K., Catalano D., Iracheta-Vellve A., Szabo G. (2015). MicroRNA-155 deficiency attenuates liver steatosis and fibrosis without reducing inflammation in a mouse model of steatohepatitis. PLoS ONE.

[40] Doukas S.G., Vageli D.P., Sasaki C.T. (2018). NF-kappaB inhibition reverses acidic bile-induced miR-21, miR-155, miR-192, miR-34a, miR-375 and miR-451a deregulations in human hypopharyngeal cells. J. Cell. Mol. Med..

[41] Mashima R. (2015). Physiological roles of miR-155. Immunology.

[42] Kim D.J., Yoon S., Ji S.C., Yang J., Kim Y.K., Lee S., Yu K.S., Jang I.J., Chung J.Y., Cho J.Y. (2018). Ursodeoxycholic acid improves liver function via phenylalanine/tyrosine pathway and microbiome remodelling in patients with liver dysfunction. Sci. Rep..

[43] Castro R.E., Ferreira D.M., Afonso M.B., Borralho P.M., Machado M.V., Cortez-Pinto H., Rodrigues C.M. (2013). miR-34a/SIRT1/p53 is suppressed by ursodeoxycholic acid in the rat liver and activated by disease severity in human non-alcoholic fatty liver disease. J. Hepatol..

[44] Sheedy F.J., Palsson-McDermott E., Hennessy E.J., Martin C., O’Leary J.J., Ruan Q., Johnson D.S., Chen Y., O’Neill L.A. (2010). Negative regulation of TLR4 via targeting of the proinflammatory tumor suppressor PDCD4 by the microRNA miR-21. Nat. Immunol..

[45] Merline R., Moreth K., Beckmann J., Nastase M.V., Zeng-Brouwers J., Tralhao J.G., Lemarchand P., Pfeilschifter J., Schaefer R.M., Iozzo R.V. (2011). Signaling by the matrix proteoglycan decorin controls inflammation and cancer through PDCD4 and MicroRNA-21. Sci. Signal..

[46] Caescu C.I., Guo X., Tesfa L., Bhagat T.D., Verma A., Zheng D., Stanley E.R. (2015). Colony stimulating factor-1 receptor signaling networks inhibit mouse macrophage inflammatory responses by induction of microRNA-21. Blood.

[47] Barnett R.E., Conklin D.J., Ryan L., Keskey R.C., Ramjee V., Sepulveda E.A., Srivastava S., Bhatnagar A., Cheadle W.G. (2016). Anti-inflammatory effects of miR-21 in the macrophage response to peritonitis. J. Leukoc. Biol..

[48] Zhu W.D., Xu J., Zhang M., Zhu T.M., Zhang Y.H., Sun K. (2018). MicroRNA-21 inhibits lipopolysaccharide-induced acute lung injury by targeting nuclear factor-kappaB. Exp. Ther. Med..

[49] Caviglia J.M., Yan J., Jang M.K., Gwak G.Y., Affo S., Yu L., Olinga P., Friedman R.A., Chen X., Schwabe R.F. (2018). MicroRNA-21 and dicer are dispensable for hepatic stellate cell activation and the development of liver fibrosis. Hepatology.

[50] Wasik U., Kempinska-Podhorodecka A., Bogdanos D.P., Milkiewicz P., Milkiewicz M. (2020). Enhanced expression of miR-21 and miR-150 is a feature of anti-mitochondrial antibody-negative primary biliary cholangitis. Mol. Med..

[51] Afonso M.B., Rodrigues P.M., Simao A.L., Gaspar M.M., Carvalho T., Borralho P., Banales J.M., Castro R.E., Rodrigues C.M.P. (2018). miRNA-21 ablation protects against liver injury and necroptosis in cholestasis. Cell Death Differ..

[52] Zhou R., Hu G., Gong A.Y., Chen X.M. (2010). Binding of NF-kappaB p65 subunit to the promoter elements is involved in LPS-induced transactivation of miRNA genes in human biliary epithelial cells. Nucleic Acids Res..

[53] Wu N., McDaniel K., Zhou T., Ramos-Lorenzo S., Wu C., Huang L., Chen D., Annable T., Francis H., Glaser S. (2018). Knockout of microRNA-21 attenuates alcoholic hepatitis through the VHL/NF-kappaB signaling pathway in hepatic stellate cells. Am. J. Physiol Gastrointest. Liver Physiol..

[54] Castro R.E., Ferreira D.M., Zhang X., Borralho P.M., Sarver A.L., Zeng Y., Steer C.J., Kren B.T., Rodrigues C.M. (2010). Identification of microRNAs during rat liver regeneration after partial hepatectomy and modulation by ursodeoxycholic acid. Am. J. Physiol Gastrointest. Liver Physiol..

[55] Rodrigues P.M., Afonso M.B., Simao A.L., Borralho P.M., Rodrigues C.M.P., Castro R.E. (2015). Inhibition of NF-kappaB by deoxycholic acid induces miR-21/PDCD4-dependent hepatocellular apoptosis. Sci. Rep..

[56] Shah S.A., Volkov Y., Arfin Q., Abdel-Latif M.M., Kelleher D. (2006). Ursodeoxycholic acid inhibits interleukin beta 1 and deoxycholic acid-induced activation of NF-kappaB and AP-1 in human colon cancer cells. Int. J. Cancer.

