# Supplementary material for: Suppression of Hepatic PPARα in Primary Biliary Cholangitis Is Modulated by miR-155
Source: Cells. 2022 Sep 15;11(18):2880. doi: 10.3390/cells11182880 (PMC9496720; doi:10.3390/cells11182880)
Supplement: Supplementary file 1 [file cells-11-02880-s001.zip › cells-1852355-supplementary.pdf]

Suplement

Overexpression of miR-21 is known to suppress inflammation by targeting programmed cell death 4 (PDCD4) and phosphatase and tensin homologue (PTEN), therefore, we investigated the expression of these target genes in cells transfected with miR-21 Mimic. In contrast to NHC, in the HepG2 cell line, we observed a substantial inhibition of PDCD4 (p=0.0001 vs. controls) and PTEN (p= 0.03 vs. controls).

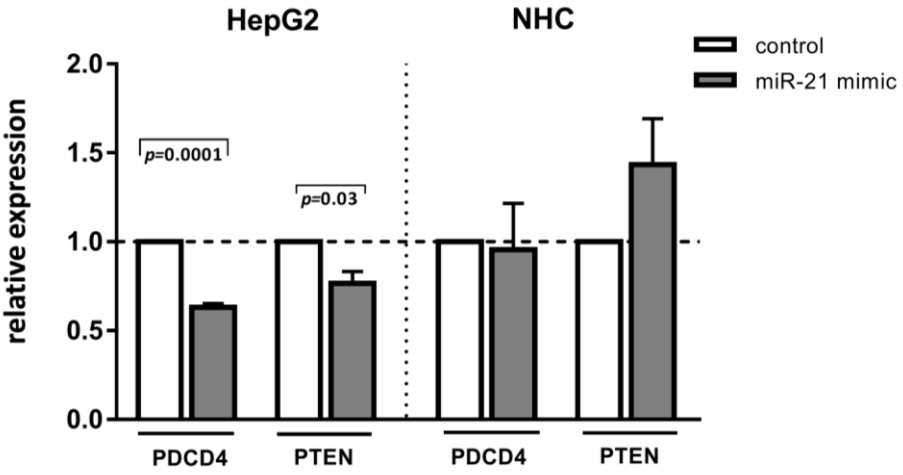

Figure S1. Programmed cell death 4 (PDCD4) and phosphatase and tensin homologue (PTEN) expression after miR-21 activation. Each experiment was repeated at least three times. Levels of gene expression were normalised to the reference 18S RNA. Bars indicate the mean ± SEM.
